# Operative Management of OSAS in a Complex Case of Proteus Syndrome

**DOI:** 10.1155/2015/137589

**Published:** 2015-06-23

**Authors:** Elena Cantone, Michele Cavaliere, Giovanni Castagna, Anna Marino, Luigi Del Vecchio, Maurizio Iengo

**Affiliations:** ^1^Department of Neuroscience, Reproductive and Odontostomatologic Science, Section of ENT, “Federico II” University, 80131 Naples, Italy; ^2^CEINGE-Biotecnologie Avanzate Scarl, 80131 Naples, Italy

## Abstract

Obstructive sleep apnea syndrome (OSAS) is a common disorder in childhood with high prevalence in syndromic subjects with craniofacial malformations. Proteus Syndrome (PS) is a rare hamartoneoplastic disorder associated with disproportionate and asymmetric overgrowth of body parts and hypertrophy or malformation of lymphatic tissues, such as palatine tonsils. We report a case of a 12-year-old boy diagnosed with Proteus Syndrome (PS) and suffering from OSAS due to asymmetric palatine tonsillar hypertrophy, treated with partial resection of left tonsil. To avoid the risk of a general anesthesia and remove only the obstructive portion of the palatine tonsil bipolar radiofrequency-induced thermotherapy (RFITT) under local anesthesia was performed. Recovery of the obstructive respiratory disease was obtained. To our knowledge, this is the first case reported in the literature of partial tonsillar resection performed in a patient with PS suffering from OSAS under local anesthesia.

## 1. Introduction

Proteus Syndrome (PS) is a complex hamartoneoplastic disorder consisting of disproportionate and asymmetric overgrowth of body parts, malformations of venous and lymphatic capillaries, and abnormal growth regulation of adipose tissue [1]. It was first described by Cohen and Heyden in 1979, but only in 1983 Rudolf Wiedemann, a German pediatrician, named it Proteus syndrome for a Greek sea-God, who could assume many forms to escape capture [1–3].

PS is extremely rare, with an estimated prevalence of approximately 1 : 1.000.000 being more common among males at a ratio of 1.9 : 1 [2]. So far, approximately 200 cases have been reported in the literature [1].

Notably, the risk of tumors, most of which are benign, is higher in PS than in general population and lipomas are the commonest [1–3]. Clinical features typically start between 1 and 18 months and develop over time. The postnatal, progressive, and asymmetric overgrowth occurs in a mosaic pattern.

Although the exact cause of PS remains unclear, it might be due to somatic alterations of a gene leading to mosaic effects and lethal if mutations are carried in nonmosaic manner. Recently, a mosaic somatic mutation of the oncogene AKT1 has been identified in more than 90% of individuals meeting diagnostic criteria [3]. AKT1 pathway is a key mediator of signal transduction from tyrosine kinase receptor to growth promoting and apoptosis-inhibiting factors. AKT inactivates through phosphorylation proapoptotic factors BAD, procaspase-9, and FOXO transcription factor, all involved in the expression of proapoptotic genes. In this way, AKT inhibits cellular death and promotes cellular survival and tissue growth. Furthermore, constitutive activation of AKT1 through Ser473 and Thr308 phosphorylation seems to underlie the oncogenic mechanism found in PS [2]; however, specific genetic diagnostic criteria have not been found yet.

From a clinical point of view, PS belongs to the group of rare hamartomatous disorders associated with hypertrophy or malformation of lymphatic tissues [3]. It is characterized by partial gigantism of hands, feet, or both, plantar hyperplasia, hemangiomas, lipomas, varicosities, verrucous epidermal nevi, macrocephaly, cranial exostosis, and asymmetry of the limbs because of long bone overgrowth.

Diagnostic criteria are classified in two groups: general and specific [4]. General criteria are mandatory, whereas specific criteria are classified into three subgroups: A, B, and C (Table 1). One feature of subgroup A or two features of B, or else three features of C, must be present to confirm the diagnosis [1, 3].

The differential diagnosis (Table 2) of PS must be done with neurofibromatosis type I (macrocephaly, café-au-lait spots, subcutaneous neurofibromas), Bannayan-Zonana syndrome (macrocephaly, craniofacial abnormalities), and other disorders presenting with hemihyperplasia, such as the Beckwith-Wiedemann syndrome (BWS), which is an overgrowth disorder with increased risk of embryonal tumors, such as Wilms tumor, hepatoblastoma, neuroblastoma, and rhabdomyosarcoma [1, 3, 5]. However, no consensus criteria for clinical diagnosis of BWS have been defined. All in all, the key finding in PS is the distortion of the skeletal architecture, whereas most of the other overgrowth syndromes show a proportionate overgrowth with preservation of the general skeletal architecture [1].

This paper reports a rare case of PS with asymmetric palatine tonsillar hypertrophy suffering from OSAS and treated with partial resection of the left tonsil under local anesthesia. To our knowledge, this is a unique case reported in the literature.

## 2. Case Presentation

**Figure 1:**
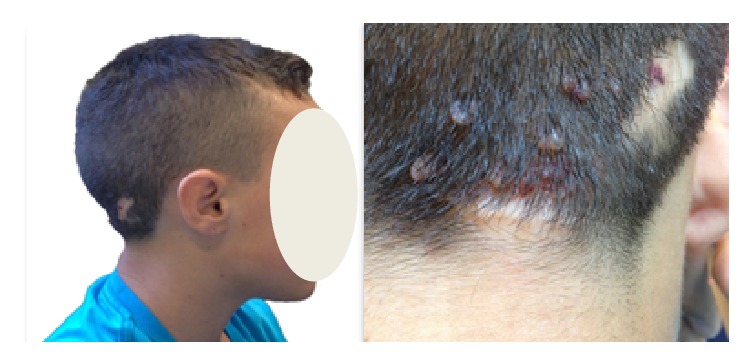
Dolichocephaly and retronuchal angiomas.

**Figure 2:**
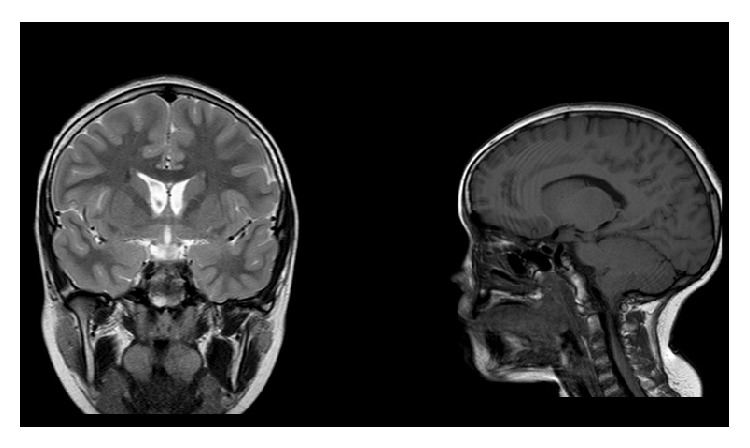
MRI.

A 12-year-old boy diagnosed with PS presented to our ENT Unit with a history of snoring and impaired swallowing. He had all of the general mandatory criteria for PS, and one feature of subgroup B (linear epidermal nevus) and three features of subgroup C (lipomas, vascular malformations, and facial phenotype) of specific criteria. In particular, he had macrocephaly, dolichocephaly, frontal bossing, and high stature for age. Some years before the child underwent surgical excision of multiple nape lipomas, whereas some angiomas in different areas were not removed (Figure 1), the ENT evaluation revealed a grade II adenoidal hypertrophy [6] and asymmetric enlargement of the palatine tonsils with a prevalence of the left one, corresponding to a Friedman grade IV with a severe narrowing of the upper airway. Laboratory tests revealed normal renal and liver functions, as well as red and white cells count. To exclude the presence of malignancy of the left tonsil, a magnetic resonance (MR) contrast image of head and neck was performed. It confirmed the presence of benign tonsillar enlargement (Figure 2) as well as vascular brain malformations, Arnold-Chiari type I, and fluid collection in prevertebral region. For this reason, the patient underwent chest computed tomography (CT) scan that showed a chylous collection in paramediastinal area, close to the lung pleura and encompassing the left subclavian and common artery.

Clinical history, symptoms, and signs suggested an OSAS, so the young patient underwent clinical and diagnostic evaluation for sleep performance. His mother filled out the Sleep Disturbances Scale for Children (SDSC), a questionnaire used for school aged children made of 26 items subdivided into six disorder subscales [7], whose score showed a severe grade of disturbance. His Epworth Sleepiness Scale score was 13/24 and his body mass index (BMI) was 27.84 kg/m^2^. Furthermore, the nocturnal polysomnography showed a severe OSAS, reporting an obstructive apnea-hypopnea index (AHI) of 28,3 with an oxygen saturation SpO2(%) nadir of 89%. Given the severity of the obstructive sleep apnea and the anesthetic risk related to mediastinal scenario, we decided to perform a partial resection of left tonsil under local anesthesia, using bipolar radiofrequency-induced thermotherapy (RFITT) [8] and taking the sample for histological examination. Written consent was obtained before surgery, explaining risks linked to the intervention and alternative therapies.

**Figure 3:**
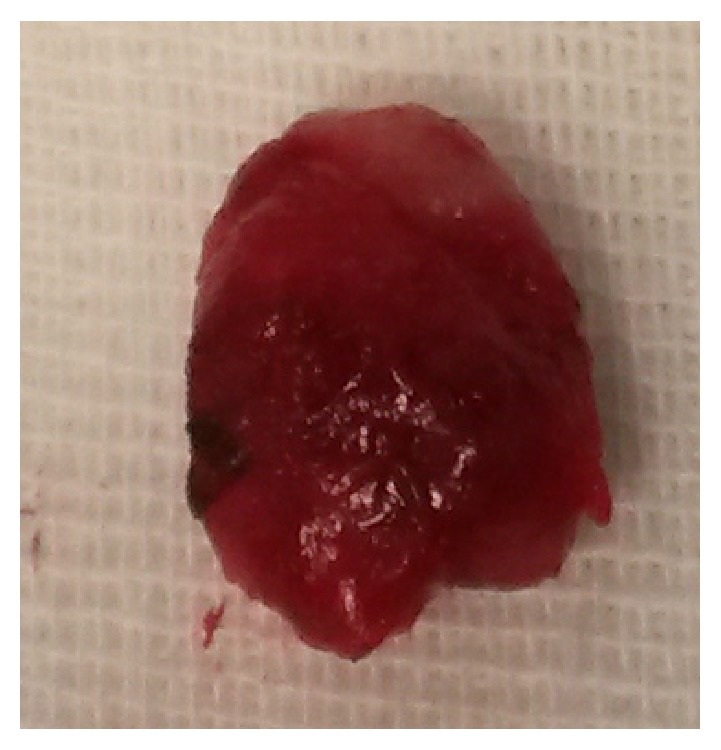
Surgical specimen.

**Figure 4:**
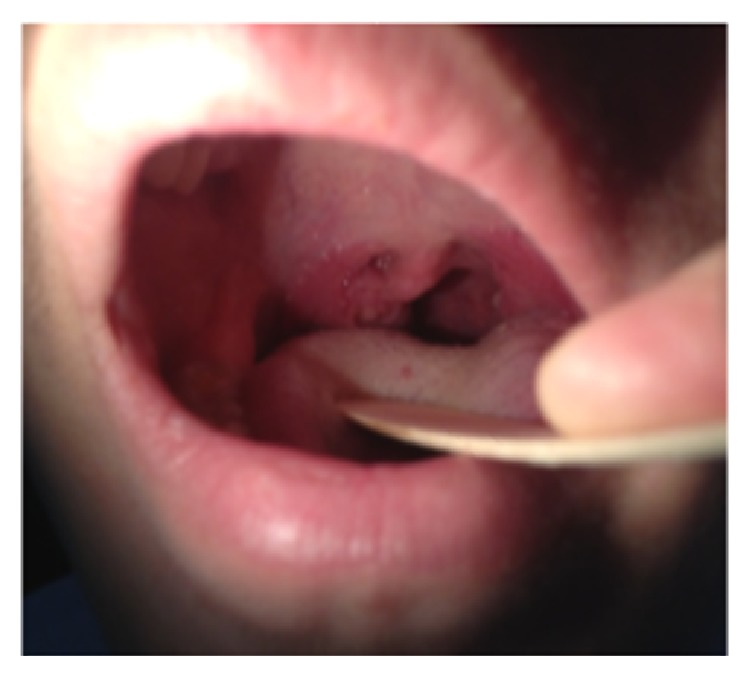
ENT evaluation after RFITT.

Histopathology confirmed the benign follicular hyperplasia (Figure 3). In addition, we performed a peripheral blood and tissue cytofluorometric exam that did not show quantitative anomalies in T, B, and NK lymphocytes. Postoperative course was regular without any complication and the patient was discharged on the same day. The postoperative follow up (Figure 4) showed improvement in symptoms, in questionnaire, and, what is most important, in the polysomnography performed 3 months after surgery that revealed an AHI of 2,4.

## 3. Discussion

In children, sleep-disordered breathing (SDB) ranges from primary snoring, through upper airway resistance, to OSAS [7]. Reports of the prevalence of habitual snoring in children ranged from 3.2% to 12.1%, and estimates of OSAS ranged from 0.7% to 10.3% [9] and even higher in syndromic children and in those with craniofacial malformations [10]. Since OSAS is common in children and is associated with significant sequelae, early diagnosis is crucial.

Diagnosis of OSAS in children is based on the sleep history, physical examination, and polysomnographic findings.

The most common cause of pediatric OSAS is adenotonsillar hypertrophy [10] but the presence of asymmetric hypertrophic tonsils, in adults as well as in children, can indicate a malignant disease. Furthermore, lymphoma is the most common head and neck malignancy in children, and palatine tonsils asymmetry is the most frequent clinical manifestation of tonsillar lymphoma [11].

We report a rare case of PS young patient with asymmetric tonsillar hypertrophy who complained with severe OSAS and difficulty in swallowing. Since the risk of tumors is higher in PS than in general population [1–3] and PS patients can present abnormalities such as narrowed airway, suboptimal positioning due to skeletal malformations, spine deformities, propensity for thromboembolism, and respiratory diseases that can lead to anaesthesiologic complications, we decided to perform surgery under local anaesthesia. Furthermore, in our patient the presence of a chylous collection in paramediastinal area should complicate surgery.

Previous studies reported asymmetric tonsillar enlargement in PS patient with OSAS treated with tonsillectomy under general anesthesia [12], but this is the first case of partial tonsillar resection under local anaesthesia. A retrospective study concludes that partial tonsillar resection is as effective as tonsillectomy for the long-term treatment of children suffering from OSAS due to hypertrophic tonsils [13]. On this basis, we opted for partial unilateral tonsillar resection using bipolar radiofrequency-induced thermotherapy (RFITT), a surgical procedure aimed to remove only the obstructive portion of the palatine tonsil (Figure 3) [8]. This technique is characterized by less postoperative pain, quicker recover, and fewer complications than tonsillectomy [8]. In addition, our patient after surgery showed significant improvement of polysomnographic values.

We believe that the partial tonsillar resection represents a therapeutic option in some selected cases of syndromic patients suffering from OSAS related to tonsillar hypertrophy, giving the possibility of both treating the sleep apnea and excluding the malignant nature of the sample.

In conclusion, the prognosis for an individual with PS is based on the location and degree of the overgrowth and the presence or absence of significant complications. A multidisciplinary approach is needed for detection and management of complications. Furthermore, PS patients require regular physical and imaging examination to recognize complications and tumors and timely operations that result in a part or complete excision of the lesion when the progressive overgrowth leads to significant loss of function [13]. Overall, in case of OSAS due to tonsillar hypertrophy, a partial resection under local anesthesia could successfully be performed to avoid the risk of a general anesthesia.

## Figures and Tables

**Table 1:** Revised Proteus syndrome diagnostic criteria [4].

General criteria	Mosaic distribution
	Progressive course
	Sporadic occurrence

Specific criteria	*Subgroup A *
	(1) Cerebriform connective tissue nevus
	*Subgroup B *
	(1) Linear epidermal nevus
	(2) Asymmetric, disproportionate overgrowth of limbs, skull, external auditory canal, vertebrae, or viscera (spleen/thymus)
	(3) Specific tumors before 2nd decade of life, as bilateral ovarian cystadenomas or monomorphic parotid adenomas
	*Subgroup C *
	(1) Dysregulation of adipose tissue: lipomas, regional lipohypoplasia
	(2) Vascular malformations (capillary, venous, and/or lymphatic)
	(3) Bullous pulmonary disease
	(4) Facial phenotype with long face, dolichocephaly, downslanted palpebral fissures, low nasal bridge, wide or anteverted nares, and open mouth at rest

For the diagnosis of Proteus syndrome all general criteria and one feature of subgroup A or two features of subgroup B or three features of subgroup C must be satisfied.

**Table 2:** Differential diagnosis.

Syndrome	Characteristics
Neurofibromatosis I	Macrocephaly, café-au-lait spots, subcutaneous neurofibromas
Bannayan-Zonana syndrome	Macrocephaly, craniofacial abnormalities
Beckwith-Wiedemann syndrome	Hemihyperplasia, embryonal tumors
Klippel-Trenaunay syndrome	Venular-venosa-linfática malformations
Proteus syndrome	Disproportionate and asymmetric overgrowth of body parts, malformations of venous and lymphatic capillaries, and abnormal growth regulation of adipose tissue
